# Recovery of metallo-tolerant and antibiotic resistant psychrophilic bacteria from Siachen glacier, Pakistan

**DOI:** 10.1371/journal.pone.0178180

**Published:** 2017-07-26

**Authors:** Muhammad Rafiq, Muhammad Hayat, Alexandre M. Anesio, Syed Umair Ullah Jamil, Noor Hassan, Aamer Ali Shah, Fariha Hasan

**Affiliations:** 1 Department of Microbiology, Quaid-i-Azam University, Islamabad, Pakistan; 2 Department of Microbiology, Abdul Wali Khan University, Mardan, Pakistan; 3 Bristol Glaciology Centre, School of Geographical Sciences, University of Bristol, Bristol, United Kingdom; 4 Department of Earth and Environmental Sciences, Bahria University, Islamabad, Pakistan; Emory University School of Medicine, UNITED STATES

## Abstract

Cultureable bacterial diversity of previously unexplored Siachen glacier, Pakistan, was studied. Out of 50 isolates 33 (66%) were Gram negative and 17 (34%) Gram positive. About half of the isolates were pigment producers and were able to grow at 4–37°C. 16S rRNA gene sequences revealed Gram negative bacteria dominated by Proteobacteria (especially γ-proteobacteria and β-proteobacteria) and Flavobacteria. The genus *Pseudomonas* (51.51%, 17) was dominant among γ- proteobacteria. β-proteobacteria constituted 4 (12.12%) *Alcaligenes* and 4 (12.12%) *Janthinobacterium* strains. Among Gram positive bacteria, phylum Actinobacteria, *Rhodococcus* (23.52%, 4) and *Arthrobacter* (23.52%, 4) were the dominating genra. Other bacteria belonged to Phylum Firmicutes with representative genus *Carnobacterium* (11.76%, 2) and 4 isolates represented 4 genera *Bacillus*, *Lysinibacillus*, *Staphylococcus* and *Planomicrobium*. Most of the Gram negative bacteria were moderate halophiles, while most of the Gram positives were extreme halophiles and were able to grow up to 6.12 M of NaCl. More than 2/3 of the isolates showed antimicrobial activity against multidrug resistant *S*. *aureus*, *E*. *coli*, *Klebsiella pneumonia*, *Enterococcus faecium*, *Candida albicans*, *Aspergillus flavus* and *Aspergillus fumigatus* and ATCC strains. Gram positive bacteria (94.11%) were more resistant to heavy metals as compared to Gram negative (78.79%) and showed maximum tolerance against iron and least tolerance against mercury.

## Introduction

Glaciers are a huge mass of moving ice that runs slowly over the land. Generally, glaciers are stable bodies of ice that consist mostly of re-crystallized snow that displays evidence of depressed slope or outward movement due to gravity. In a glacier bionetwork, microorganisms play significant role in cycling of carbon, subglacial weathering [1] and other nutrients. For example, snow algae act as primary producer that sustain heterotrophic population on glaciers, such as copepods, insects, fungi and bacteria [2]. Organic carbon is trapped deep in the glacial ice, microbes metabolize it and form methane [3], a greenhouse gas. This conversion of carbon to methane could be of significance in climate change [4]. Subglacial microbes perform mineral weathering [5] and make available minerals and other nutrients for fellow life forms. Moss can survive for centuries underneath glaciers, and recolonizes land as the ice retreats [6]. There is a potent connection between geochemical signatures in subglacial materials and the metabolic processes occurring in that environment. Studies of glaciers and other habitats of permanent snow and ice have shown a diverse range of cold tolerant organisms [2, 7]. Previously glacial ice was considered biologically inactive or life-entrapping medium that collects and preserves microorganisms that are deposited through rain or snow [8]. Scientists have discovered that glaciers can be a favorable environment to support active and diverse communities of micro- as well as macrobiota [9, 10]. The presence of bacteria in polar and non-polar glaciers have been reported by many researchers through both culture-dependent and culture-independent techniques [11–13]. Dormant and vegetative forms of bacteria exist under ice of glacier and are adapted to this unique ecosystem by one or more diverse mechanisms [14]. Comparison of geographically distinct glaciers worldwide have shown a great variation in microbial biomass and community structure [15–17] which is mainly controlled by climatic and environmental factors, including geographic location [1, 18] wind direction, wind speed, light intensity, and availability of nutrients and liquid water [19]. There is some limited evidence of biogeographic effects on the distribution of microorganisms in the geographically distinct glaciers [15–17, 20] and the factors driving the dynamics of microbial community in glacial systems are still not clear.

Many scientists have reported a number of bacterial species including *Pedobacter himalayensis* [21], *Exiguobacterium indicum* [22], *Dyadobacter hamtensis* [23], *Leifsonia pindariensis*, *Bacillus cecembensis* [24], *Cryobacterium roopkundense* [25], *Cryobacterium pindariense* [26] and *Paenibacillus glacialis* [27] from snow, water, soil and sediments of glaciers located in Himalaya. Baghel et al [13] reported the psychrotrophic proteolytic bacteria from Gangotri Glacier, Western Himalaya, India. Bacterial populations in Roopkund Glacier, Himalayan mountain ranges, were studied by Branda et al. [28]. They found Actinobacteria as the predominant class, followed by β- proteobacteria. Actinobacteria are potent producers of antimicrobial compounds and thus can have dominant role in generating a stress on other microbial life. Bacterial diversity of soil samples from Drass, India a coldest place after Siberia, was explored and screened for various hydrolytic enzymes [29]. Phylogenetic analysis revealed 40 different bacteria, grouped into three major phyla, Proteobacteria, Actinobacteria and Firmicutes differentiated into 17 different genera. These isolates were also investigated for production of hydrolases at 4–30°C. All the isolates secreted one or the other hydrolytic enzyme, i.e. esterase (90%), lipase (80%), protease (32.5%), amylase (20%) and cellulase (17.5%). These results indicate that culturable bacteria in soil of Drass could serve as an ideal candidate for enzyme bioprospecting.

Dumping of non-biodegradable waste in large quantities and the use of arms and ammunition have considerably affected the ecosystem of the region [30]. The troops on the glacier dump the waste in the crevasses of the glacier. About 40% of the waste at the glacier is plastic and metals including cobalt, cadmium and chromium that affects water of the Shyok River (which enters Indus River near Skardu). The water of Indus is used for drinking and irrigation [31, 32]

The aim of the present study was to isolate bacteria from Siachen glacier, Pakistan, and to characterize the strains on basis of different physiological characteristics and determine antibiotic and metal resistance and their antimicrobial activity.

## Materials and methods

### Sample collection

The area from where the samples were collected was not inside the private land. The sampling sites were accessed with support of people of the local community, and the procedures did not involve any disturbance to endangered or protected species of animals or vegetation. Three types of samples (glacial ice, glacial melt water and glacial sediment) were collected from Siachen glacier, Pakistan (35°25′16″N 77°06′34″E/35.421226°N77.109540°E Google map coordinates of the glacier, not specifically of the sampling site) in sterile bottles and transported to the laboratory at the Department of Microbiology, Quaid-i-Azam University, Islamabad. The samples were processed for isolation of bacterial strains following standard microbiological protocols. The samples were stored at low temperature till further analysis. Temperature and pH of sample site were also recorded.

### Reagents and chemicals

Media, sodium chloride, metal salts, H_2_SO_4_, HCl and NaCl were obtained from Sigma Chemicals Co. (St. Louis, MO, USA). Antibiotic discs were from (Oxoid, Limited, Basingstoke, Hampshire, England).

### Total viable count

Total viable count of the samples was determined by calculating colony forming units (CFU) per ml or g for each sample. About 200 μl of melted ice, sediment or glacial water samples were spread on R2A medium. Water and ice samples were diluted to 1:10 by adding 1 ml sample in tubes containing 9 ml sterile saline, while sediment sample was diluted by adding 1 g in 10 ml normal saline. All the plates were incubated at two different temperatures 4°C and 15°C for about 60 days. Number of culturable bacteria was estimated by counting the average colony formation units (CFU/ml or g) on individually R2A agar plate. Characteristic bacterial colonies with visually different morphologies were chosen and subcultured to obtain pure colonies.

### Isolation and identification of bacteria

The isolation and characterization of culturable bacterial isolates wwas performed according to Zang et al. [33]. About 50 distinct colonies were identified according to phenotypic properties like (colony morphology, growth properties pigment production), physiological characteristics (pH, temperature range, sodium chloride tolerance) and 16S rRNA gene sequencing.

In the current study, a total of all the 50 isolates were checked for antibacterial and antifungal activity, metal tolerance and resistance to commonly used antibiotics. The isolates were consistently cultured on LB agar and R2A agar and stored as glycerol stocks at -70°C.

### DNA extraction, sequencing and phylogenetic analysis

The bacterial DNA was extracted according to protocol previously described by Shivaji et al. [34]. The 16S rRNA sequencing was done by Macrogen Inc. Seoul, Korea. The sequences obtained were further evaluated by comparing the nucleotide sequences available in NCBI database [35]. The evolutionary history was inferred on method based on the Tamura-Nei model [36]. The phylogenetic tree was constructed in MEGA software [36]. at the bootstrap value 1000.

### Deposition of accession numbers in NCBI

Sequences of all isolates described in this study were deposited in NCBI (National Center for Biotechnology Information) under the accession numbers from KX128918 to KX128967, as the following:

HS1 KX128918, HS2 KX128919, HS3 KX128920, HS4 KX128921, HS5 KX128922, HS6 KX128923, HS7 KX128924, HS8 KX128925, HS9 KX128926, HS10 KX128927, HS11 KX128928, HS13 KX128929, HS14 KX128930, HS16 KX128931, HS17 KX128932, HS18 KX128933, HS19 KX128934, HS21 KX128935, HS22 KX128936, HS23 KX128937, HS24 KX128938, HS25 KX128939, HS26 KX128940, HS27 KX128941, HS28 KX128942, HS29 KX128943, HS30 KX128944, LS1 KX128945, LS2 KX128946, LS3 KX128947, LS4 KX128948, LS5 KX128949, LS7 KX128950, LS8 KX128951, LS15 KX128952, LS16 KX128953, LS17 KX128954, LS18 KX128955, LS19 KX128956, LS20 KX128957, LS22 KX128958, LS23 KX128959, LS24 KX128960, LS25 KX128961, LS26 KX128962, LS27 KX128963, LS29 KX128964, LS30 KX128965, LS35 KX128966, LS36 KX128967.

### Antibiotic resistance

Antimicrobial susceptibility was evaluated through disk diffusion method following the guidelines of the Clinical and Laboratory Standards Institute CLSI, 2013. A total of 9 antibiotics representing different classes; colistin sulphate (CT 10 μg); sulfamethoxazole/trimethoprim (SXT 23.75/1.25 μg), clindamycin (DA 2 μg), Ofloxacin (OFX 5 μg), imipenem (IMI 10 μg), cefotaxime (CTX 30 μg), nalidixic acid (NA 30 μg), Vancomycin (VA 30 μg) and Methicillin (ME 5 μg) were used for determination of antibiotic resistance.

### MAR index

Multiple Antibiotic Resistance index was calculated using formula:

MARindex=a/b,

where “a” represents the number of antibiotics to which the isolates were resistant, while “b” represents the total number of antibiotics used.

### Metal tolerance

Metal tolerance was checked by prepared stock solutions (4000 ppm) of each heavy metal. The minimum inhibitory concentration (MIC) of heavy metals was determined using LB medium (Sigma) containing Cd^+2^, Cr^+3^, Hg^+2^, Fe^+3^, Ar^+3^ and Ni^+3^ (5–1300 ppm). The metals were supplemented as CdCl_2_. 2H_2_O, CrCl_3_, HgCL_2_, FeCl_2_, ArCl_3_ and NiCl_3_. The isolates were considered resistant when the MIC values exceeded that of *E*. *coli* and *S*. *aureus* used as a control.

### Screening for antimicrobial activity

The antimicrobial activity was determined by spot on lawn assay. Briefly, the cell suspensions of bacteria, *Candida* and fungal spores were prepared according to 0.5 McFarland standard and were spread on Muller-Hinton agar and ~ 5 μL of each isolate was spot inoculated and the plates were incubated at 15°C for 72 to 96 hours. A clear zone of inhibition around the indicator organism indicated the antagonistic effect.

### Indicator microorganisms

In order to screen the isolates for antimicrobial activity, drug resistant bacterial, fungal and bacterial ATCC cultures were used as test organisms. Multidrug resistant isolates including *S*. *aureus*, *E*. *coli*, *Klebsiella pneumoniae*, *Enterococcus faecium*, *Candida albicans*, *Aspergillus flavus* and *Aspergillus fumigatus*, and ATCC strains of *S*. *aureus* (ATCC6538), *E*. *coli* (ATCC10536) and *Pseudomonas aeruginosa* (ATCC27853) were used as indicator microorganisms. The MDR (multi drug resistant) strains were obtained from Medical Microbiology Laboratory, Department of Microbiology, Quaid-i-Azam University, Islamabad.

## Results

### Recovery of bacteria

Out of three samples, the richest source of bacteria in terms of CFU/g or mlwas glacial sediment, followed by melt water and ice, respectively. The number of viable cells was slightly higher at 15°C as compared to 4°C (Table 1). On the basis of colony morphology, a total of 50 bacterial strains were isolated from all samples of Siachen glacier. A total of 23 isolates were obtained at 4°C and 27 at 15°C. On the basis of different colony morphology, 24 isolates were recovered from glacial sediment, 14 from melt water and 12 from glacial ice (Table 1).

**Table 1:** Viable count of study samples in term CFU/gm or ml.

Sample	pH	Temp (°C)	CFU/gm or ml at different Incubation Temp		No. of different Isolates
			4°C	15°C	
Glacial ice	7	-2	2.34 x 10^5^	7.01 x 10^5^	12
Glacial melt water	7	2	9.92 × 10^5^	3.65 x 10^6^	14
Glacial sediment	7	1	3.73 × 10^8^	1.53 × 10^9^	24

### Microscopic, morphological and physiological identification

Identification and classification of bacterial isolates was done according to Zhang et al. [33]. The isolates were placed in two distinct groups on the basis of microscopic analysis. Gram negative bacterial isolates [66% (33)] were more prevalent than Gram positive [34% (17)] bacteria. Almost half of the isolates were observed to produce pigments.

All the isolates could grow at temperature ranging from 4°C to 37°C, however, these isolates fail to grow at 45°C. Among Gram negative bacteria, 81.81% (27 isolates) did not show growth at 37°C, while 6.06% (2 isolates) failed to grow at 15°C and 6.06% (2 isolates) were unable to grow at 4°C. Among Gram positive bacteria 41.17% (7 isolates) were unable to grow at 37°C, 5.88% (1 isolate) and 11.76% (2 isolates) could not grow at 15 and 4°C, respectively.

All the strains showed a remarkable level of tolerance to increasing concentrations of NaCl [from 0.9 to 36% (0.14–6.12 M)]. The NaCl tolerance ranging from 0.15–1.33 M (0.9–8%) was observed in 14 (42.42%) Gram negative bacteria, 15 (45.45%) isolates showed growth at NaCl ranging from 1.33–3.4 M (8 to 20%), while 4 (12.12%) isolates showed tolerance to 3.4–6.12 M (20–36%) concentration of NaCl. Of Gram positive bacteria a single bacterial isolate (5.88%) showed growth at 0.9–2% (0.15–0.34 M NaCl, 3 (17.64%) isolates showed growth at salt concentration 1.8–3.4 M (8–20%) while, 13 (76.47%) isolates tolerated 22 to 36% (3.74–6.12 M) NaCl concentration (Table 2).

**Table 2:** Morphological and microscopic charactarization of study isolates.

Isolates	Sample type	Gram Reaction	Morphology of the colony	Pigment production	Temperature range (°C)			NaCl tolerance	
					4	15	37	Range tested (%)	Optimum range (%)
LS1	Ice	-ive	White large, convex, diplobacilli/tetrods	✘	✓	✘	✘	0.9–16	2–10
LS 2	Ice	-ive	Orange, flate dry, diplobacilli	✓	✓	✓	✘	0.9–18	2–10
LS 3	Ice	-ive	Greyish white, large, Bacilli scattered/diplo	✓	✓	✓	✘	0.9–18	2–6
LS4	Ice	-ive	White shiny, coccobacilli in scattered form	✘	✓	✓	✘	0.9–14	0.9–2
LS5	Ice	-ive	White, large and viscous, coccobacilli	✘	✓	✘	✘	0.9–14	2–6
LS15	Ice	-ive	white transparent, fluidy coccobacilli	✘	✓	✓	✘	0.9–18	2–10
LS20	Sediment	-ive	Orange colour, Diplococci	✓	✓	✓	✓	0.9–22	2–16
LS23	Melt Water	-ive	Large yellowish fluidy, thin diplobacilli	✓	✓	✓	✘	0.9–26	2–16
LS24	Melt Water	-ive	Extra-large off white rounded, scattered cocci	✘	✓	✓	✘	0.9–18	2–10
LS26	Sediment	-ive	White rounded scattered bacilli	✘	✓	✓	✘	0.9–14	2–6
LS30	Sediment	-ive	Off-white, raised, scattered cocci	✘	✓	✓	✘	0.9–18	2–10
LS35	Sediment	-ive	White circular, coccobacilli 2/3 cell	✘	✓	✓	✘	0.9–12	2–6
LS36	Sediment	-ive	White small rounded colony, coccobacilli	✘	✓	✓	✓	0.9–22	2–14
HS2	Sediment	-ive	Large off-white shiny, mucoid thick bacilli	✘	✓	✓	✘	0.9–4	0.9–2
HS3	Sediment	-ive	Light yellow raised cantered, scattered bacilli	✓	✓	✓	✘	0.9–12	2–6
HS4	Sediment	-ive	Off-white, like fry egg, bacilli	✘	✓	✓	✘	0.9–36	2–28
HS7	Sediment	-ive	Large orange colour, scattered bacilli	✓	✓	✓	✘	0.9–2	0.9
HS8	Sediment	-ive	Off-white dark opaque, bacilli	✘	✓	✓	✘	0.9–2	0.9
HS9	Sediment	-ive	Yellowish, medium and opaque, bacilli	✓	✓	✓	✓	0.9–8	0.9–2
HS10	Sediment	-ive	Rough surface like fried egg dry diplobacilli	✓	✓	✓	✓	0.9–4	0.9–2
HS11	Sediment	-ive	Light yellow with unique margin bacilli	✓	✓	✓	✘	0.9–8	0.9–2
HS13	Sediment	-ive	White small transparent, 2/3 pair of cells	✘	✓	✓	✘	0.9–8	2–4
HS14	Sediment	-ive	White shiny, medium sized bacilli	✘	✓	✓	✘	0.9–4	0.9
HS17	Melt water	-ive	Large yellow, shiny opaque, scattered bacilli	✓	✓	✓	✘	0.9–8	2–4
HS18	Melt water	-ive	Orange colour, bacilli in scattered form	✓	✓	✓	✘	0.9–10	2–6
HS19	Melt water	-ive	Large flat, orange colour bacilli scattered form	✓	✓	✓	✘	0.9–4	1–2
HS21	Melt water	-ive	Large yellowish flate and transparent bacilli	✓	✓	✓	✘	0.9–8	0.9–2
HS22	Melt water	-ive	Pale yellow, large flate transparent, bacilli	✓	✘	✓	✘	0.9–6	1–2
HS23	Melt water	-ive	Deep orange colour, thin coccobacilli	✓	✓	✓	✓	0.9–8	2–6
HS24	Melt water	-ive	Extra-large, raised convex fluidy water bacilli	✘	✓	✓	✘	0.9–10	1–2
HS25	Melt water	-ive	Large bright yellow, opaque, bacilli	✓	✘	✓	✘	0.9–8	0.9–2
HS26	Melt water	-ive	Off white large rough surface dry, bacilli	✘	✓	✓	✘	0.9–12	1–4
HS28	Ice	-ive	Light yellow, raised opaque thick coccobacilli	✓	✓	✓	✓	0.9–14	2–10
LS7	Ice	+ive	Deep yellow, large sticky, rods	✓	✓	✘	✘	0.9–14	2–6
LS8	Ice	+ive	Large white, thick rods, in pair of 2/4 cells	✘	✓	✓	✓	0.9–26	2–18
LS16	Sediment	+ive	Yellow, medium sized diplobacilli	✓	✓	✓	✓	0.9–22	2–16
LS17	Sediment	+ive	White, thick diplobacilli	✘	✓	✓	✘	0.9–22	2–16
LS18	Sediment	+ive	Brownish white, Bacilli	✘	✓	✓	✘	0.9–22	2–14
LS19	Sediment	+ive	White, cocci or diplococci	✘	✓	✓	✓	0.9–18	2–14
LS22	Melt Water	+ive	Small orange to light yellow, diplobacilli	✓	✓	✓	✘	0.9–22	2–16
LS25	Melt Water	+ive	Large white colony, diplobacilli	✘	✓	✓	✘	0.9–22	2–14
LS27	Sediment	+ive	Yellow rounded streptococci	✓	✓	✓	✘	0.9–26	2–20
LS29	Sediment	+ive	Purple colour colony, scattered cocci	✓	✓	✓	✓	0.9–18	2–12
HS1	Sediment	+ive	White, medium sized, cocci	✘	✓	✓	✓	0.9–36	2–26
HS5	Sediment	+ive	Yellowish colony, thick bacilli	✓	✘	✓	✓	0.9–36	2–24
HS6	Sediment	+ive	Light yellow colony, bunch/ chain form	✓	✘	✓	✓	0.9–36	2–26
HS20	Melt water	+ive	White, large and opaque, staphylococci	✘	✓	✓	✘	0.9–2	0.9
HS27	Ice	+ive	Yellow, medium sized, short/thick bacilli	✓	✓	✓	✓	0.9–36	2–28
HS29	Ice	+ive	Dark orange, diplococci	✓	✓	✓	✓	0.9–22	2–14
HS30	Ice	+ive	Small white, bacilli	✘	✓	✓	✓	0.9–22	2–10

Most of the Gram negative isolates belonged to proteobacteria with predominance of γ- proteobacteria and β-proteobacteria. The genus *Pseudomonas* (17 isolates) dominated the γ- proteobacteria group, while the other genera belonging to this class were *Psychrobacter* (2 isolates) and *Acinetobacter* (1 isolate). The Class β-proteobacteria constitutes 4 isolates of *Alcaligenes* and 4 isolates of *Janthinobacterium*, however, a single isolate of genus *Afipia* belongs to class α-proteobacteria. Similarly, Flavobacteria belonging to phylum Bacteroides was represented by *Flavobacterium* (2 isolates), *Chryseobacterium* (1 isolate) and 1 novel isolate (Table 3). Gram positive isolates belonging to phylum and class Actinobacteria with dominating genera of *Rhodococcus* (4 isolates), *Arthrobacter* (4 isolates), *Leucobacter* (2 isolates) and *Brevibacterium* (1 isolate), while Firmicutes with representative genera *Carnobacterium* (2 isolates) and 4 isolates representing 4 genera including *Bacillus*, *Lysinibacillus*, *Staphylococcus* and *Planomicrobium*.

**Table 3:** Comparative study of bacteria isolates resistant to different antibiotics.

Antibiotics	Gram negative (n = 33)			Gram positive (n = 17)		
	S	I	R	S	I	R
Imipenem	48.48	0.0	51.51	35.29	0.0	64. 70
Oflaxacin	75.75	0.0	24.24	88.23	0.0	11.76
Sulfamethoxazole/ trimethoprim	87.87	0.0	12.12	94.12	5.88	0.0
Cefotaxime	54.54	0.0	45.45	11.76	11.76	76.47
Clindamycin	45.45	9.09	45.45	29.41	11.76	58.82
Colistin sulphate	69.7	9.09	21.21	NA	NA	NA
Nalidixic Acid*	72.	12.12	15.15	NA	NA	NA
Methicillin	NA	NA	NA	35.29	5.88	58.82
Vancomycin	NA	NA	NA	17.65	17.64	64.70

Key: **S–Sensitive, R–Resistant, I–Intermediate**

### Phylogenetic analysis

**Fig 1:**
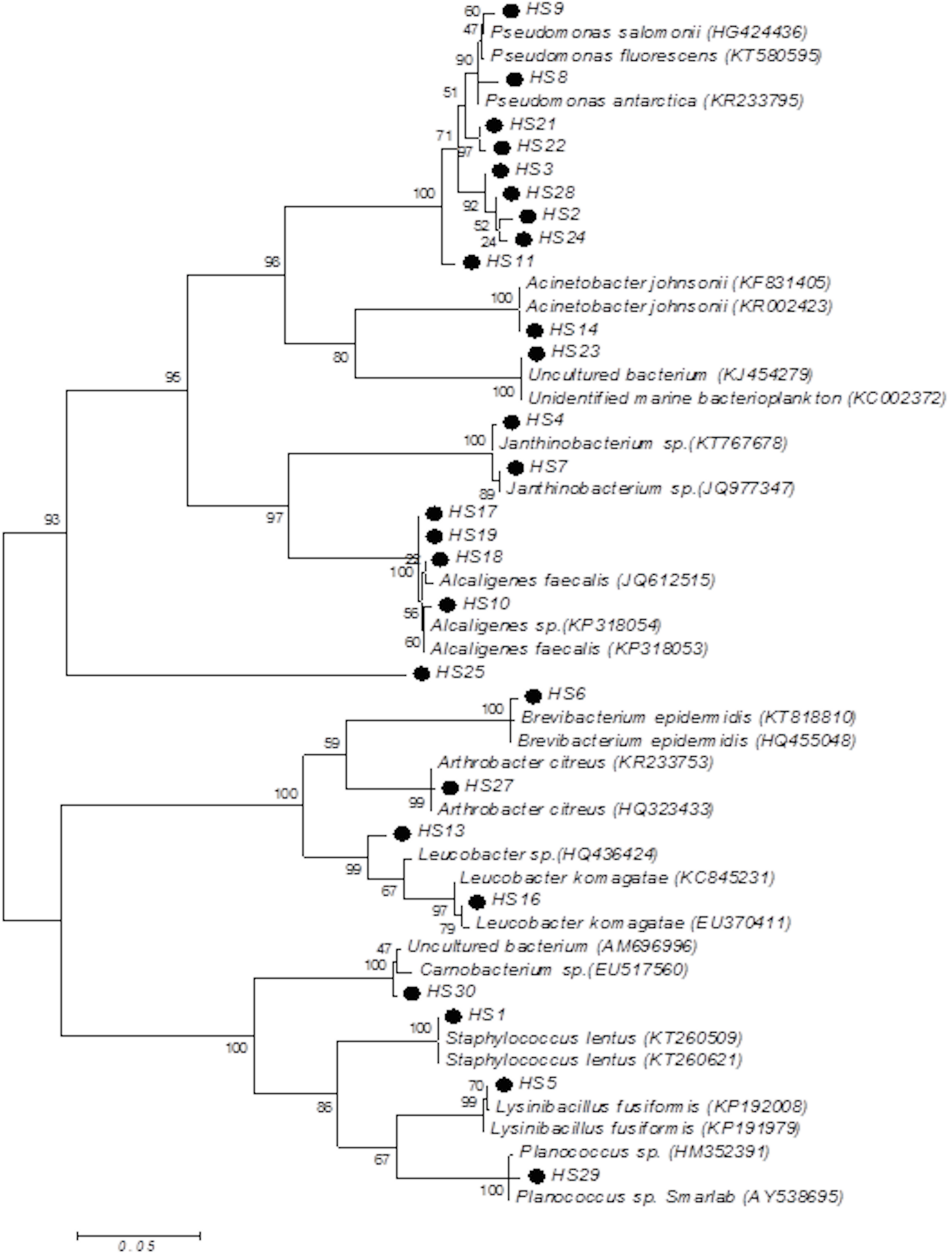
Phylogenetic tree of the study isolates HTS (15°C) by Maximum Likelihood method constructed in MEGA 6 software.

**Fig 2:**
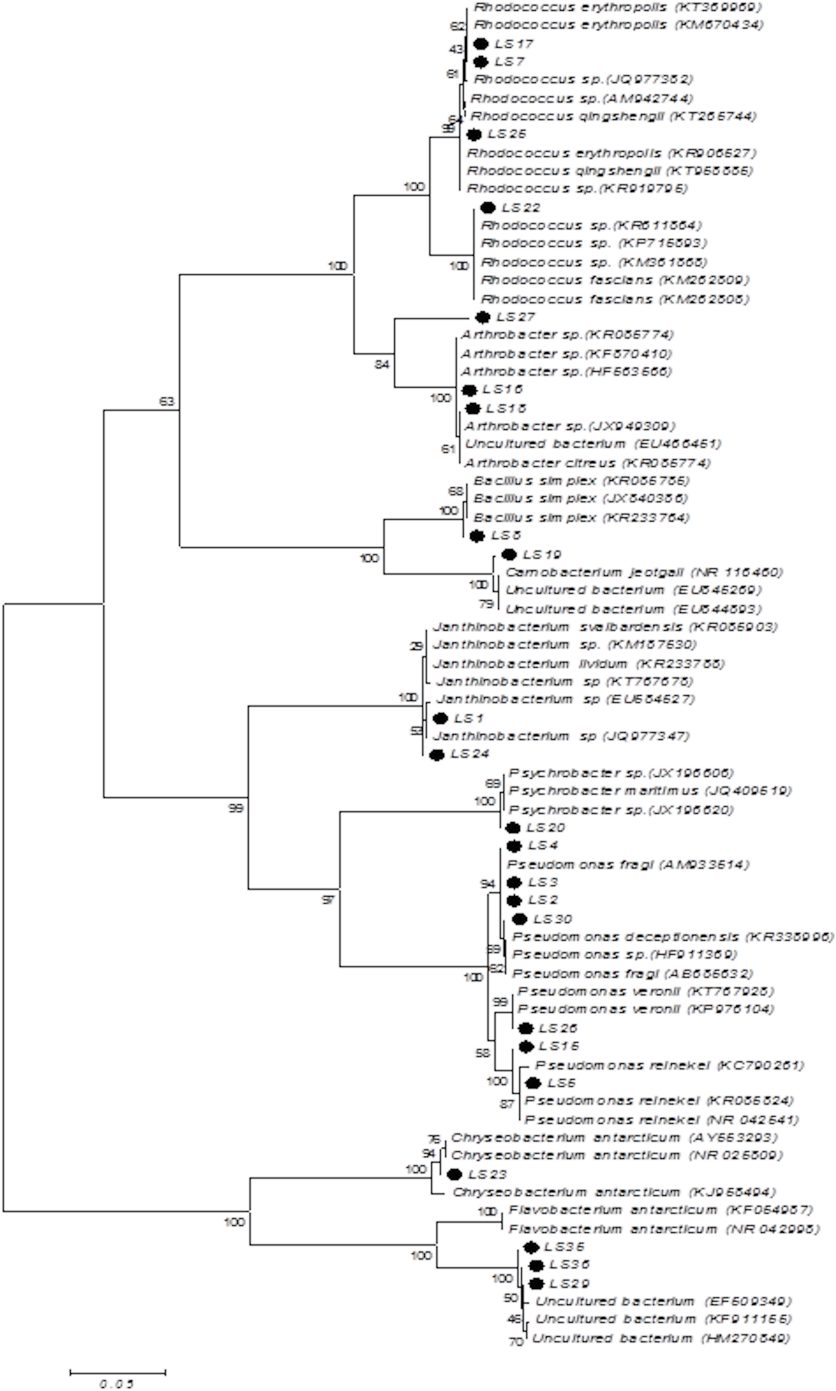
Phylogenetic relationship analysis of study isolates (LTS) by Maximum Likelihood method in MEGA 6 software.

The evolutionary history was inferred by using the Maximum Likelihood method based on the Tamura-Nei model 42. The tree with the highest log likelihood (-4713.0244) is shown. The percentage of trees in which the associated taxa clustered together is shown next to the branches. Initial tree(s) for the heuristic search were obtained automatically by applying Neighbor-Join and BioNJ algorithms to a matrix of pairwise distances estimated using the Maximum Composite Likelihood (MCL) approach, and then selecting the topology with superior log likelihood value. The tree is drawn to scale, with branch lengths measured in the number of substitutions per site. The analysis involved 53 nucleotide sequences. Codon positions included were 1^st^ + 2^nd^ + 3^rd^ + Noncoding. All positions containing gaps and missing data were eliminated. There were a total of 593 positions in the final dataset. Evolutionary analyses were conducted in MEGA6. The phylogenetic tree of both High and Low temperature isolates are given in Fig 1 & Fig 2.

### Antibiotic resistance

A total of 9 antibiotics (5 broad spectrum antibiotics, 2 antibiotics against Gram positive and 2 against Gram negative bacteria) were used to evaluate the antibiotic resistance pattern of all the isolates. Interestingly, these isolates showed some varying resistance to all antibiotics used in the current study.

Among Gram negative study isolates, more resistance was found against imipenem (51.51%, 17 isolates) then cefotaxime (CTX) and clindamycin (DA) each 45.45%, (15 isolates) followed by resistance to Ofloxacin (OFX) 24.24%, 8 isolates. Only 21.21%, (7 isolates) showed resistance to colistin sulphate (CT), 15.15%, 5 isolates were resistant to Nalidixic acid, while 12.12%, 4 isolates were resistant to sulfamethoxazole/trimethoprim (SXT). The Gram positive isolates were highly resistant to majority of the antibiotics. Resistance to cefotaxime (CTX) was more prevalent in 76.47% (13 isolates) followed by resistance to imipenem and Vancomycin in 64.70%, 11 strains each. Resistance to CTX and Methicillin was observed in 58.82% (10 isolates). Only (11.76%, 2 isolates showed resistance to Ofloxacin while all the isolates were sensitive to combination of sulphamethaxazole/trimethoprim (Table 3)

### Multiple antibiotic resistance (MAR) index

Multiple antibiotic resistance index was determined.Among Gram negative bacteria, 78.79% (26 isolates) showed multiple antibiotic resistance, 9.09% (3 isolates) showed resistance to 1 antibiotic and 12.12% (4 isolates) were sensitive to all antibiotics (Table 4). About 94.11% (16 isolates) Gram positive bacteria showed multiple antibiotic resistance, while 5.89% (1 isolate) showed resistance to a single antibiotic (Table 5).

**Table 4:** Antibiotic resistance and production of antimicrobial compounds in Gram negative bacteria isolated from Siachen Glacier.

Isolates		Nearest phylogenetic species*	Antibiotic resistance and production of antimicrobial compounds in Gram negative bacteria																		
			Antibiotic Resistance and sensitivity pattern							MAR index	Antibacterial and antifungal activity										
			IMI	CTX	CT	NA	SXT	OFX	DA		*S*.A*		*E*.C*	*P*.A*	*C*.*A*	*A*.*F*	*A*.*Fl*	*S*.*A*	*E*.*C*	*K*.*P*	*Ent*.
Gammaproteobacteria																					
LS 2	*Pseudomonas* sp.		R	R	I	R	S	S	I	0.71	-		1	2	**-**	**-**	-	-	-	1	1
LS 3	*Pseudomonas fragi*		S	S	I	S	S	S	R	0.28	-		-	-	**-**	**-**	NA	NA	NA	NA	NA
LS4	*Pseudomonas fragi*		S	R	S	S	S	S	R	0.28	-		-	-	**-**	**-**	NA	NA	NA	NA	NA
LS5	*Pseudomonas reinekei*		S	R	R	S	R	R	S	0.57	1		-	-	*-*	*-*	NA	NA	NA	NA	NA
LS15	*Pseudomonas* sp		S	R	R	I	S	S	R	0.57	-		-	-	**-**	**-**	NA	NA	NA	NA	NA
LS20	*Psychrobacter* sp.		S	S	S	I	S	S	R	0.28	-		-	-	**-**	**-**	NA	NA	NA	NA	NA
LS26	*Pseudomonas veronii*		S	R	S	S	S	S	S	0.14	-		-	-	-	-	NA	NA	NA	NA	NA
LS30	*Pseudomonas fragi*		R	R	S	S	S	S	R	0.42	-		-	-	2	1	1	NA	NA	NA	NA
HS2	*Pseudomonas fragi*		S	S	S	S	S	S	R	0.14	-		-	-	-	-	NA	NA	NA	NA	NA
HS3	*Pseudomonas deceptionensis*		R	S	S	S	S	S	R	0.28	-		-	-	-	-	NA	NA	NA	NA	NA
HS8	*Pseudomonas antarctica*		R	S	S	I	R	S	S	0.42	-		-	-	-		NA	NA	NA	NA	NA
HS9	*Pseudomonas salomonii*		R	S	S	S	R	S	S	0.28	1		1	1	1	1	1	1	1	1	1
HS11	*Pseudomonas frederiksbergensis*		S	S	S	S	S	S	S	0.00	1		1	1	1	-	-	-	1	1	-
HS14	*Acinetobacter johnsonii*		S	S	S	S	S	S	S	0.00	1		2	1	2	2	2	1	-	1	1
HS21	*Pseudomonas* sp.		R	S	R	S	S	R	R	0.57	1		2	2	2	-	-	1	1	-	-
HS22	*Pseudomonas arsenicoxydans*		S	S	R	I	S	R	S	0.42	-		-	-	-	-	1	1	NA	NA	NA
HS23	*Psychrobacter* sp.		R	S	S	S	S	R	S	0.28	-		-	-	-	-	NA	NA	NA	NA	NA
HS24	*Pseudomonas* sp.		R	S	S	R	R	S	I	0.57	-		-	-	-	-	NA	NA	NA	NA	NA
HS26	*Pseudomonas* sp.		S	R	S	S	S	S	S	0.14	-		-	-	-	-	NA	NA	NA	NA	NA
HS28	*Pseudomonas* sp.		R	R	S	S	S	S	R	0.42	-		-	-	-	-	NA	NA	NA	NA	NA
Betaproteobacteria																					
LS1	*Janthinobacterium* sp.		R	S	I	S	S	**S**	S	0.28		-	-	-	**-**	**-**	NA	NA	NA	NA	NA
LS24	*Janthinobacterium* sp.		R	R	S	S	S	S	R	0.42		2	2	1	1	-	2	1	-	-	-
HS4	*Janthinobacterium lividum*		S	S	S	S	S	S	S	0.00		-	-	-	-	-	NA	NA	NA	NA	NA
HS7	*Janthinobacterium* sp.		S	R	S	S	S	S	I	0.28		-	-	-	-	-	NA	NA	NA	NA	NA
HS10	*Alcaligenes* sp.		R	R	S	R	S	S	S	0.42		1	1	-	2	2	2	-	-	1	-
HS17	*Alcaligenes* sp. HT4-MRL		S	R	R	S	S	R	S	0.42		1	1	1	1	-	1	-	1	1	-
HS18	*Alcaligenes faecalis*		R	S	S	S	S	R	S	0.28		1	1	1	2	-	1	2	-	1	-
HS19	*Alcaligenes* sp. HT4-MRL		R	S	R	S	S	S	R	0.42		1	1	-	1	-	-	1	-	-	-
Alpha proteobacteria																					
HS25	*Afipia* sp.		S	S	S	S	S	S	S	0.00		-	-	-	-	-	NA	NA	NA	NA	NA
Flavobacteria																					
LS23	*Chryseobacterium antarcticum*		S	R	S	S	S	S	R	0.28		-	-	-	*-*	*-*	NA	NA	NA	NA	NA
LS29	Uncultured bacteria gb|EF509349.1|		R	S	S	R	R	R	S	0.57		2	2	1	-	-	-	-	-	1	1
LS35	*Flavobacterium antarcticum*		R	R	R	R	S	R	R	0.85		2	2	-	-	1	-	1	1	1	-
LS36	*Flavobacterium antarcticum*		R	R	S	S	S	S	R	0.42		2	2	2	1	-	2	1	1	2	-

Key: *S*.A*, *Staphylococcus aureus; E*.C*, *E*. *coli; P*.A*, *P*. *aeruginosa; C*.*A*, *Candida albicans; A*.*F*, *Aspergillus fumigatus; A*.*Fl*, *A*. *flavus; S*.*A*, *S*. *aureus; E*.*C*, *E*.*coli; K*.*P*, *Klebsiella pneumoniae*, *En*, *Enterococcus*. R resistant, S sensitive and NA not applicable

**Table 5:** Antibiotic resistance, mutliple antibiotic resistant (MAR) index and antimicrobial activity of Gram positive bacterial isolates.

Isolates	Nearest phylogenetic neighbour or belonging to the species*	Antibiotic resistance and antimicrobial compounds production in Gram Positive bacteria																					
		Resistance to antibiotics								*MAR Index*	Antimicrobial and antifungal compounds												
		IMI	OFX	SXT	CTX	DA	VA	ME			*S*.A*	*E*.C*	*P*.A*	*S*.*A*	*E*.*C*	*K*.*P*	*Ent*	*A*.*Fl*	*A*.*F*			*C*.*A*	
**Actinobacteria**																							
LS7	*Rhodococcus* sp.	R	S	S	R	R	R	R		0.71	-	1	-	NA	NA	NA	NA	-	-			-	
LS17	*Rhodococcus erythropolis*	R	S	S	R	S	I	R		0.57	-	-	-	NA	NA	NA	NA	-	-			-	
LS22	*Ryyhodococcus* sp.	S	S	S	R	R	R	R		0.57	-	-	-	NA	NA	NA	NA	-	-			-	
LS25	*Rhodococcus* sp.	S	S	S	R	R	R	R		0.57	-	-	-	1	-	-	-	2	2			1	
LS16	*Arthrobacter* sp.	S	S	S	I	S	R	S		0.14	1	-	-	NA	NA	NA	NA	-	-			-	
LS18	*Arthrobacter* sp.	R	S	S	R	R	R	R		0.71	-	-	-	NA	NA	NA	NA	-	-			-	
LS27	*Arthrobacter* sp.	R	S	S	R	R	R	R		0.71	-	-	-	NA	NA	NA	NA	-	-			2	
HS27	*Arthrobacter citreus*	R	S	S	I	S	S	R		0.42	-	-	-	NA	NA	NA	NA	-	-			-	
HS13	*Leucobacter*	R	S	S	R	S	R	R		0.57	-	2	1	-	-	2	1	-	-			1	
HS20	*Leucobacter* sp.	S	R	S	R	I	S	R		0.57	-	-	-	NA	NA	NA	NA	-	-			-	
HS6	*Brevibacterium* sp.	R	S	I	R	R	I	R		0.85	-	-	-	NA	NA	NA	NA						
**Bacilli**																							
LS8	*Bacillus simplex*	R	S	S	R	R	R	R	0.71		-	2	1	-	-	-	1	-		-			-
HS5	*Lysinibacillus fusiformis*	S	R	S	R	I	I	S	0.57		-	-	-	-	-	-	-	1		1			2
LS19	*Carnobacterium pleistocenium*	R	S	S	R	S	S	S	0.28		-	-	-	NA	NA	NA	NA	-		-			-
HS30	*Carnobacterium alterfunditum*	R	S	S	S	R	R	S	0.42		-	-	-	-	-	-	-	1		1			2
HS1	*Staphylococcus lentus*	S	S	S	R	R	R	I	0.57		-	-	-	NA	NA	NA	NA	-		-			-
HS29	*Planomicrobium* sp	R	S	S	S	S	R	S	0.28		-	-	-	NA	NA	NA	NA	-		-			-

Key: *S*.A*, *Staphylococcus aureus; E*.C*, *E*. *coli; P*.A*, *P*. *aeruginosa; C*.*A*, *Candida albicans; A*.*Fl*, *A*. *flavus; A*.*F*, *Aspergillus fumigatus; S*.*A*, *S*. *aureus; E*.*C*, *E*.*coli; K*.*P*, *Klebsiella pneumoniae*, *En*, *Enterococcus*, R resistant, S sensitive and NA not applicable

### Screening for antimicrobial activity

All the 50 isolates were screened for their antimicrobial activity. Among Gram negative bacteria, 13 (39.39%) isolates showed antimicrobial activity against 4 or more than 4 test strains, while 3 distinct isolates showed inhibitory effects against 1, 2 and 3 isolates. About 3 (17.64%) Gram positive bacteria showed antimicrobial activity against 1 test strain, 3 (17.64%) showed activity against 3 and 2 (11.76%) of the isolates showed antimicrobial activity against 4 or more than 4 test strains (Tables 4 & 5).

### Metal tolerance

All isolates were screened for their tolerance against 6 different metal ions and minimum inhibitory concentration was determined. Among Gram negative bacteria the minimum inhibitory concentration of Cadmium were 651–850 ppm in (36.36%, 12 isolates), (39.39%, 13 isolates) and (54.54%, 18 isolates) showed tolerance to 651–850 ppm of chromium and nickel respectively, 27.27%, 9 isolates tolerate arsenic level ranging from 851–1050, and (27.27%, 9 isolates tolerate iron level greater than 1050 ppm, however the MIC level in case of Mercury was ≤ 50 in 18 (54.54%) isolates. Of Gram positive bacteria 8, 47.05% isolates showed tolerance to cadmium and 6 (35.29%) showed tolerance to nickel, in the range of 651–850 ppm. Minimum inhibitory concentration of chromium was 451–651 ppm in 5 isolates while 5 (29.41%) isolates showed tolerance to 851–1050 ppm of cadmium. A minimum inhibitory concentration of arsenic and iron ranging from (851–1050 ppm) was noted in 6 (35.29%) and 7 (41.17%) isolates respectively. The MIC of mercury was ≤ 50 in 11 (64.70%) while the highest tolerable level was observed ˂ 120 ppm in mercury. Comparative analysis of both Gram positive and Gram negative bacterial strains is given in Table 6.

**Table 6:** Tolerance of Gram negative and Gram positive bacteria to varying concentrations of metal ions.

Representative groups	Metal	Heavy metal concentration (μL/mL or PPM)						
		≤ 50	51–250	251–450	451–650	651–850	851–1050	>1050
**Gram negative bacterian = 33**	*Cadmium*	All	All	18.18, 6	24.24, 8	36.36, 12	12.12, 4	9.09, 3
	*Chromium*	All	All	24.24, 8	30.31, 10	39.39, 13	6.06, 2	None
	*Arsenic*	All	9.09, 3	15.15, 5	24.24, 8	21.21, 7	27.27, 9	6.06, 2
	*Nickel*	All	12.12, 4	9.09, 3	18.18, 6	54.54, 18	3. 03, 1	None
	*Iron*	All	All	15.15, 5	12. 12, 4	21.21, 7	24.24, 8	27.27, 9
	*Mercury*	54.54, 18	45.46, 15	None	None	None	None	None
**Gram positive bacterian = 17**	*Cadmium*	All	All	All	23.52, 4	47.05, 8	17.64, 3	11.76, 2
	*Chromium*	All	All	17.64, 3	29.41, 5	23.52, 4	29.41, 5	None
	*Arsenic*	All	All	5.88, 1	23.52, 4	29.41, 5	35.29, 6	5.88, 1
	*Nickel*	All	11.76, 2	23.52, 4	17.64, 3	35.29, 6	11.76, 2	None
	*Iron*	All	All	5.88, 1	17.64, 3	29.41, 5	41,17, 7	17.64, 3
	*Mercury*	64.70, 11	35.29, 6	None	None	None	None	None

## Discussion

To our understanding, this is the first report regarding the diversity of antibiotic producing, metallo- tolerant and antibiotic resistant bacteria isolated from Siachen glacier. In the current research work, A total 50 islates from 3 different samples of glacial ice, melt water and sediment were recovered. Sediment had the highest number because it is known to contain numerous nutrients and have slightly higher temperature [37] as compared to ice and glacial melt water. It is also obsereved that Gram negative bacteria were more dominant and abundant as compared to Gram positive bacteria, which is in a close association with previous studies [38, 39, 40] that also reported high prevalence of Gram negative bacteria with predominance of γ-proteobacteria, α-proteobacteria and β-proteobacteria. The dominance of bacterial diversity in a particular glacier or cold environments might be due to the seasonal variation in glaciers which can counter-select the bacteria with greater adaptability. Boetius et al. [41] identified bacterial isolates with greater abundance of Gram positive bacteria which is contradictory to our finding. The predominance of Gram negative bacteria in the current study could be related to the psychrophilic nature of Gram negative bacteria, as psychrophiles have been reported to grow faster and out-compete psychrotrophic bacteria [42].

There was a significant difference in terms of growth range among Gram negative and Gram positive bacteria. Most of the Gram negative bacteria (78.78%) were able to grow at 15°C, while most of the Gram positive (58.82%) isolates were able to grow at 37°C. According to definition of Turley [43], the Gram negative bacteria can be placed among psychrophilic, and the Gram positive isolates in psychrotrophic bacteria. Previously, Carpenter et al. [44] identified bacteria from South Pole snow, all of which were true psychrophiles while Morita [45] isolated and characterised bacteria from Ellesmere Island ice as psychrotrophs. Around the year the temperature of Siachen valley remains far below 0°C (down to -41°C) to 11°C [46]. It is unclear how these bacteria survive under such a diverse conditions. However, the survival of psychrotrophic bacteria in extremely stressful conditions is due to formation of spores in Gram positive bacteria as described earlier [47–49]. These findings strongly support our study as most of the Gram positive bacteria in our study were psychrotrophic and spore formers. Spore formation might help overcome the stress conditions to low temperature, desiccation and damage of bacteria by UV [50–51]. The possible mechanism of survival in Gram negative bacteria could be associated with upregulation of desaturase genes and increase of membrane lipids like Poly-unsaturated fatty acids (PUFAs) in association with decrease in temperature [52, 53].

Our isolates also showed tolerance to different concentrations of NaCl ranging from 0.14–6.12 M (0.9 to 36%). In active ecological environments in glacial habitats, water nuclei forms inside glacier mass, the solutes around that environment diffuse to this active ecological environment and make it hypertonic [54]. The exposure of bacteria to such conditions leads to salt tolerance. In our isolates the increased tolerance could be due to this phenomenon. It is for the first time to report salt tolerance of glacial bacteria above 5.1 molar concentration, although detailed investigation and research is required to investigate the phenomenon of such a high tolerance in depth.

The current research also showed increased antibiotic resistance in our isolates. Previous investigations from pristine cold environments like ancient Siberian permafrost, alpine glacier cryoconite and non-anthropogenic alpine soil [29, 55, 56] opposed our results as bacteria from such environments have been reported to have greater sensitivity to antibiotics. However, wide distribution and multiple antibiotic resistance genes have been previously documented in different glaciers except antarctic glaciers and has been well described by transmission of migratory birds and air borne bacteria [57].

Our isolates also showed increased tolerance to various metals. Previous reports [58, 59] also supported our findings, however, these earlier studies do not include all the metals used in our studies. This is the first study of Siachen glacier, Pakistan, also include to the intrinsic property of low temperature bacteria to demonstrate metal and antibiotic resistance with antimicrobial activity.

Siachen glacier is known as the world’s highest non-polar glacier and considered as the highest and world’s biggest garbage dump, 40% of which are plastics and metals, worn out gun barrels, splinters from gun shelling, empty fuel barrels and burnt shelters, which permanently pollute glacial ice and water and leach toxins like cobalt, cadmium, chromium and other metals due to unavailability of natural biodegrading agents [60]. The heavy metal tolerance could possibly be due to these pollutants, and could possibly lead to antibiotic resistance too, as metal and antibiotic resistance is often present as co-resistance [61]. On the other hand Siachen glacier is the world’s highest warzones. The antibiotic resistance in such area could also be due to army patrolling that might harbour pathogenic or opportunistic bacteria that can transmit resistance genes to environmental bacteria. The third possible reason for antibiotic resistance is the production of antimicrobial compounds that leads to natural resistance in such bacteria.

About 40% of the isolates produced antimicrobial compounds against American Type Culture Collection (ATCC) and clinical isolates of bacteria, yeasts and molds. Our results are strongly supported by works of previous scientists [31, 62] who identified potent bacterial isolates from diverse cold habitats, a large number of which produced antimicrobial compounds. However, our results are in contrast to the research carried out by many scientists [63–66] on Antarctic, arctic, Argentine soil and marine organisms. The low temperature habitats are less explored as compared to mesophiles and data regarding antimicrobial compounds is very rare. Therefore, it is necessary to study these isolates along with neighbour glaciers, ice caps and glacial lakes to reveal the microbial compounds.

We conclude that the bacteria belonging to diverse groups were present with *Pseudomonas* as the most dominant genus, with Gram positive more abundant than the Gram negative bacteria. Low temperature adapted bacteria isolated from Siachen glacier, showed varying degree of resistance to metals and commonly used antibiotics and also they showed pronounced ability to inhibit the other ATCC as well as pathogenic bacteria. They were also moderate to extreme halophilic in nature.
